# Retraction Note: Computational simulation for MHD peristaltic transport of Jeffrey fluid with density-dependent parameters

**DOI:** 10.1038/s41598-026-48858-9

**Published:** 2026-04-20

**Authors:** M. G. Ibrahim, M. Y. Abou-zeid

**Affiliations:** 1 Department of Basic and Applied Science, International Academy For Engineering and Media Science, IAEMS, Cairo, 11311 Egypt; 2 Department of Mathematics, Faculty of Education, Ain Shams University, Heliopolis, Cairo, 11757 Egypt

Retraction of: *Scientific Reports* 10.1038/s41598-023-36277-z, published online 06 June 2023

The Editors have retracted this Article.

After publication concerns were raised regarding the description and implementation of the methods. Post-publication peer review confirmed issues with an incorrect definition of the Prandtl number, invalid model‑validation procedures, contradictory boundary‑condition formulations, and inconsistent treatment of variable density. The reference list also contains multiple references that are irrelevant to the context in which they are cited. The Editors have therefore lost confidence in the reliability of this Article.

Neither of the Authors did respond to the correspondence regarding this retraction.

